# Sex-Specific Protection of Osteoarthritis by Deleting Cartilage Acid Protein 1

**DOI:** 10.1371/journal.pone.0159157

**Published:** 2016-07-14

**Authors:** Xianpeng Ge, Susan Y. Ritter, Kelly Tsang, Ruirui Shi, Kohtaro Takei, Antonios O. Aliprantis

**Affiliations:** 1 Department of Medicine, Division of Rheumatology, Immunology and Allergy, Brigham and Women’s Hospital and Harvard Medical School, Boston, Massachusetts, United States of America; 2 Central Laboratory, Peking University School and Hospital of Stomatology, Beijing, China; 3 Department of Developmental Biology, Harvard School of Dental Medicine, Boston, Massachusetts, United States of America; 4 Molecular Medical Bioscience Laboratory, Department of Medical Life Science, Yokohama City University Graduate School of Medical Life Science, Yokohama, Japan; University of Texas Southwestern Medical Center, UNITED STATES

## Abstract

Cartilage acidic protein 1 (CRTAC1) was recently identified as an elevated protein in the synovial fluid of patients with osteoarthritis (OA) by a proteomic analysis. This gene is also upregulated in both human and mouse OA by transcriptomic analysis. The objective of this study was to characterize the expression and function of CRTAC1 in OA. Here, we first confirm the increase of CRTAC1 in cartilage biopsies from OA patients undergoing joint replacement by real-time PCR and immunohistochemistry. Furthermore, we report that proinflammatory cytokines interleukin-1beta and tumor necrosis factor alpha upregulate CRTAC1 expression in primary human articular chondrocytes and synovial fibroblasts. Genetic deletion of *Crtac1* in mice significantly inhibited cartilage degradation, osteophyte formation and gait abnormalities of post-traumatic OA in female, but not male, animals undergoing the destabilization of medial meniscus (DMM) surgery. Taken together, CRTAC1 is upregulated in the osteoarthritic joint and directly induced in chondrocytes and synovial fibroblasts by pro-inflammatory cytokines. This molecule is necessary for the progression of OA in female mice after DMM surgery and thus represents a potential therapy for this prevalent disease, especially for women who demonstrate higher rates and more severe OA.

## Introduction

Osteoarthritis (OA) is the most common joint disease and a major cause of disability and rising health care costs [1, 2]. Epidemiologic studies show that sex-differences exist in the incidence and severity of OA, and women have higher risks and tend to develop more severe OA [3–5]. Although great efforts have been made to understand the pathophysiology of OA and to develop therapeutics, current medications only target symptoms. There are no treatments available to prevent or block the progression of structural deterioration of the joint [6]. A better understanding of the biologic processes that drive OA, and in particular sex-specific mechanisms, could lead to new targets, and eventually disease modifying therapy.

OA is a disease of the entire joint organ [7, 8]. The synovial fluid (SF) bathes all the joint components, including cartilage, synovium, meniscus, ligaments intraarticular adipose tissue and bone. SF contains many proteins that maintain normal joint function, such as the biolubricant Proteoglycan 4 (i.e. Lubricin). In the OA joint, changes in the composition of the SF may promote the disease by changing its physiochemical properties and facilitating communication between tissues [9]. In an effort to understand the characteristics of human OA SF, we performed a proteomic analysis and identified 66 differentially expressed proteins in OA versus healthy SF [10]. By comparing our OA SF proteome to published gene expression profiles of OA cartilage and synovium, we identified cartilage acidic protein 1 (CRTAC1) as a concordantly up-regulated gene [10–12]. Interestingly, *Crtac1* is also greatly upregulated in mouse post-traumatic OA by transcriptomic analysis [13]. Here, we sought to define the role of CRTAC1 in OA.

CRTAC1, also called cartilage expressed protein 68 (CEP-68) or lateral olfactory tract usher substance (LOTUS), was first identified as a gene induced during chondrogenesis [14]. This glycoprotein contains an N-terminal signal sequence, 4 FG (phe-gly)-GAP (gly-ala-pro) domains, a UnvB/ASPIC domain and a C-terminal epidermal growth factor-like (EGF) Ca^2+^ binding domain [15]. CRTAC1 is highly conserved, with mouse and human CRTAC1 showing >90% identity. In humans, alternative usage of terminal exons (exons 15A and 15B) produces transcripts with (CRTAC1B, exon 15B) and without (CRTAC1A, exon 15A) the transmembrane domain. Whereas CRTAC1B is only expressed in brain, CRTAC1A is expressed in articular cartilage and lung [15]. The only functional data on CRTAC1 comes from studies in the central nervous system, where CRTAC1 was shown to be an antagonist of NOGO receptor-1 (NGR1) [16, 17]. The NOGO pathway has a well-established role in the brain and spinal cord, where the NGR1 ligand (NOGO) inhibits axon growth and regeneration [18]. CRTAC1 promotes axon growth in the brain by blocking the interaction of NGR1 and its ligands NOGO-A.

Although CRTAC1 was identified as a cartilage expressed protein 15 years ago, its function in the skeletal system remains undefined. Based on the dramatic increase of its expression in both human and mouse OA, we hypothesized that CRTAC1 mediates OA pathology and targeting this molecule may provide new therapeutic potential for this prevalent disease. In this study, we validated the increased expression of CRTAC1 in the articular cartilage of OA patients, and found that IL-1β and TNF-α upregulate *CRTAC1* in primary human articular chondrocytes and synovial fibroblasts. Importantly, deletion of *Cratc1* in female mice reduced cartilage degradation, osteophyte formation and gait abnormalities in a model of post-traumatic OA induced by surgical destabilization of medial meniscus (DMM). Thus, CRTAC1 is necessary for the progression of OA in female mice, and targeting this molecule may represent a novel therapy for OA in women.

## Materials and Methods

### Human OA samples

**Fig 1:**
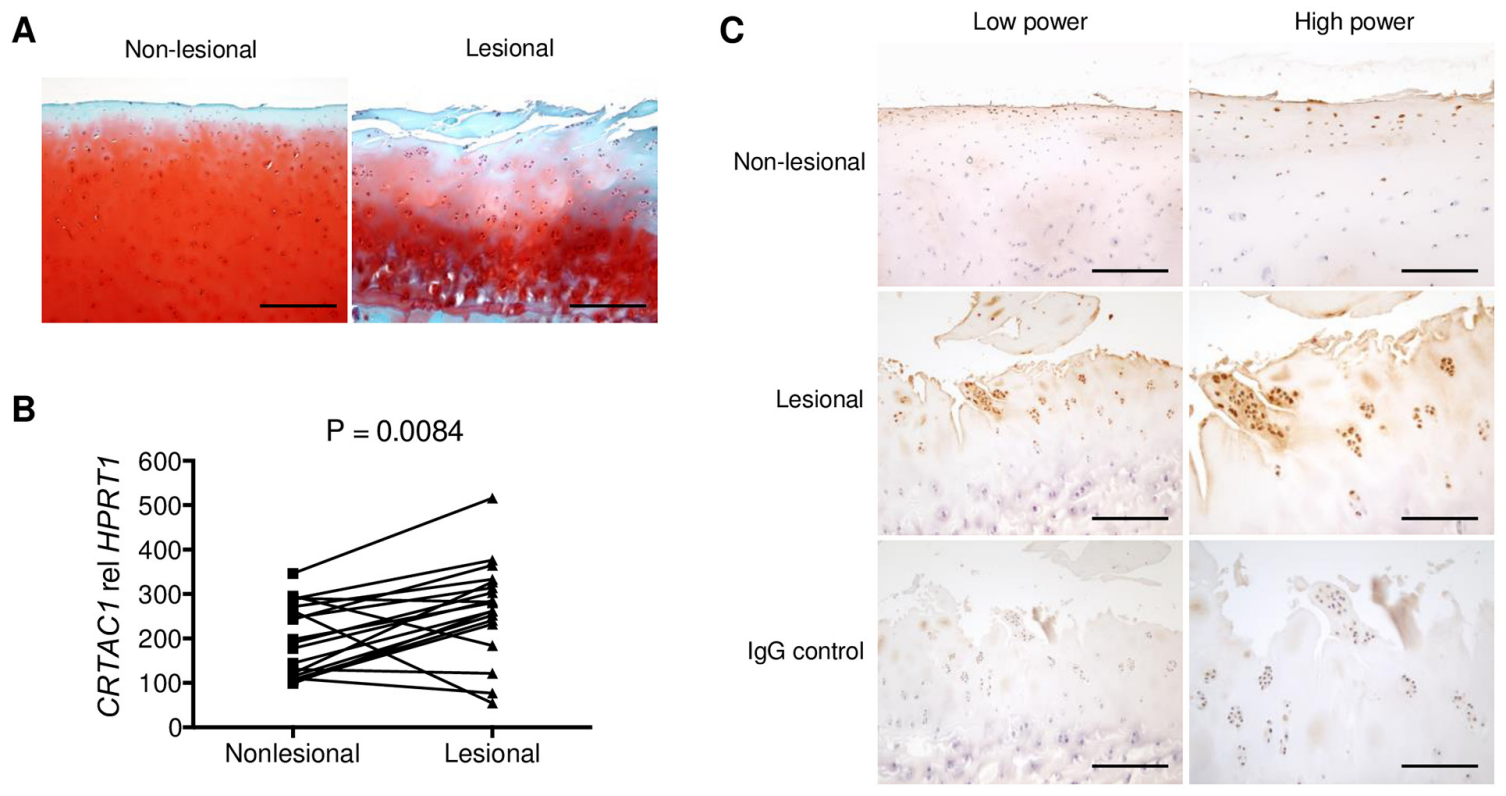
Upregulation of CRTAC1 in the articular cartilage of human osteoarthritis (OA). **A**, Representative safranin-O/fast green stain of lesional and macroscopically normal (non-lesional) articular cartilage from knee OA patients undergoing total joint replacement. Scale bars, 200 μm. **B**, Real-time PCR analysis of *CRTAC1* expression in lesional and non-lesional cartilage biopsies from advanced OA patients (n = 19). Paired two-tailed *t-test* was performed. **C**, Immunohistochemistry of CRTAC1 on lesional and macroscopically normal (non-lesional) articular cartilage from OA patients (results are representative of n = 6). Normal sheep IgG was used as a negative control. Scale bars, 200 μm (left panels), 100 μm (right panels).

For gene expression analysis and immunohistochemistry of CRTAC1, human OA samples were obtained from the lesional and non-lesional regions of articular cartilage from 19 and 6 patients with late OA undergoing total knee replacement, respectively. Histology was used to validate our choice of lesional and non-lesional articular cartilage where the former displayed hallmarks of OA including the degradation of articular cartilage and a reduction in safranin-O staining (Fig 1A). All human samples in the present study were obtained as surgical discards under a protocol (2007-P-002441) approved by the Partners Healthcare Institutional Review Board (IRB). Under this protocol, our IRB allows us to collect samples and then access associated clinical data in the medical record for two weeks, after which all identifying information is discarded. Because the samples are rapidly deidentified, patient consent was not required or obtained.

### Primary human articular chondrocytes

Human articular chondrocytes were isolated from the macroscopically non-lesional articular cartilage of OA patients undergoing total knee replacement. After rinsing tissues with HBSS, the cartilage was cut into small pieces, digested using 0.5% trypsin for 1 hr at 37°C, and subsequently 0.08% Collagenase II (Worthington Biochemicals, Lakewood, NJ) for 16 hrs. After passing the digest through a 70 μm filter, cells were plated and cultured for 3 days. All experiments were performed using P1 cells.

### Human OA synovial fibroblasts

Human knee synovium was obtained from discard surgical tissues after knee replacement for advanced OA and digested with collagenase (type IV; Worthington Biochemicals). Resulting cells were serially cultured for four passages to obtain purified synovial fibroblast populations as previously described [19]. Synovial fibroblasts were used for experiments between passages four and ten. The concentration of IL-1β and TNF-α (Thermo Fisher Scientific, Waltham, MA) used to stimulate chondrocytes and synovial fibroblasts was 10 ng/ml.

### Animals and DMM surgery

*Crtac1*^*-/-*^ mice [16] and the DMM procedure [20, 21] were previously described. Briefly, female and male *Crtac1*^*+/-*^ mice were crossed to generate *Crtac1*^*-/-*^ and wild type (*Crtac1*^*+/+*^) littermate control mice. Mice at 10 weeks of age were anesthetized using a cocktail of Ketamine, Xylazine and Acepromazine, and the right hind knee was prepared for dissection. The medial meniscal ligament was visualized and transected, and the incision was closed with sutures. Mice in the sham group were similarly treated without transecting the medial meniscal ligament. Buprenorphine 0.05–0.1 mg/kg was administrated subcutaneously during the surgical procedure for analgesia. After the surgery, Buprenorphine was administrated q 8–12 h for 48 hours, and mice were monitored daily for 5 days for mobility, grooming, posture, activity level and gait to ensure that the mouse is not experiencing undue post-operative pain. The incision was monitored to make sure that is dry, clean, and intact and shows no signs of infection. Mice showing signs of infection, hunching, shivering, decreased activity, redness or swelling or an exudate present at the surgical site or the knee joint would be euthanized. All mice were on C57BL6 background and housed in the animal facility of the Harvard T.H. Chan School of Public Health on a 12-hour light/dark cycle with ad libitum access to water and normal chow. All procedures in this study were approved by the Harvard Medical School Institutional Animal Care and Use Committee.

### Histology and immunohistochemistry

Mice undergoing DMM surgery were euthanized 8 and 16 weeks after surgery by CO2 narcosis and secondary cervical dislocation. Joints were decalcified and embedded as described [20]. Serial sections of 4 μm thickness were cut and collected at 5 sections per slide. We got about 25–30 slides from each joint. Sections at 80 μm intervals were taken for safranin-O/fast green stain, and two blinded observers scored 6 slides from each joint. The scoring system for articular cartilage was a semiquantitative scale of 0–6, looking at cartilage features including loss of safranin-O staining, fibrillation of cartilage and frank cartilage denudation down to the level of calcified cartilage [20, 22]. To evaluate osteophyte formation, a modified 0–3 scoring paradigm was used according to the thickness and maturity of osteophytes: 0 = normal, 1 = mild, 2 = moderate and 3 = severe changes [23, 24]. Both maximum and summed score were reported.

For CRATC1 immunohistochemistry, lesional and non-lesional OA articular cartilage from patients undergoing joint replacement were fixed in 4% PFA for 48 hrs and decalcified in 14% EDTA for 2 weeks. After antigen retrieval with 2.5% hyaluronidase (Sigma, St. Louis, Missouri), sections were incubated with sheep anti-human CRTAC1 antibody (R&D) or normal sheep serum for 2 hrs at room temperature, followed by biotinylated rabbit anti-sheep secondary antibody for 1 hr. Color was developed using the DAB kit (Vector Laboratories).

### Real-time polymerase chain reaction (PCR)

Lesional and non-lesional articular cartilage tissues from OA patients were grinded thoroughly in liquid nitrogen, and RNA was extracted by combining the Trizol reagent and the RNeasy mini kit (Qiagen). RNA of articular chondrocytes and synovial fibroblasts stimulated with IL-1β and TNF-α was isolated using Trizol reagent. All RNA samples were treated with the RNase-Free DNase Set (Qiagen), and equal amounts (1–2 μg) were used for reverse transcriptase reaction using random primers (AffinityScript QPCR cDNA Synthesis Kit). qPCR reactions were performed on an Mx3005P qPCR system (Agilent Technologies) using Fast SYBR Green Master Mix (Life Technologies). The expression level of CRTAC1 was normalized to the housekeeping genes hypoxanthine phosphoribosyltransferase (HPRT) or glyceraldehyde 3-phosphate dehydrogenase (GAPDH). The following primer sequences were used: *CRTAC1*, forward 5’-TGTCCAGGATGTTACCGTTCC-3’, reverse, 5’-AGCTGGGTGGGATTACTGTCA-3’; *HPRT*, forward, 5’-TGGACAGGACTGAACGTCTTG-3’, reverse, 5’-CCAGCAGGTCAGCAAAGAATTTA-3’; *GAPDH*, forward, 5’-GGAGTCCACTGGCGTCTTCAC-3’, reverse, 5’-GAGGCATTGCTGATGATCTTGAGG-3’.

### Gait analysis

Gait analysis was performed at 16 weeks after DMM surgery using the DigiGait Imaging System (Mouse Specifics, Inc., Boston, MA), as previously described [25, 26]. Briefly, animals walked on a motor-driven treadmill with a transparent treadmill belt. A high-speed digital video camera was mounted below the transparent treadmill belt. Digital video images of the underside of the animals were captured at up to 150 frames per second. Each animal was allowed to explore the treadmill compartment for ~1 minute with the motor speed set to 0. Approximately 3–6 sec of videography were collected for each mouse to provide more than 7 sequential strides. Only video segments in which the animal walked with a regularity index of 100% were used for image analyses. The walking speed used was 30 cm/s. Gait data are collected and pooled from left and right hind limbs, respectively.

### 3′Rapid amplification of cDNA ends (RACE)

The 3′-end of mouse *Crtac1* was amplified using the method of classic RACE [27]. The sequences of gene specific primers (GSP) used for mouse *Crtac1* were: GSP1, 5’-GAATGCATCCAGTTCCCATT-3’; GSP2, 5’-TGTGTCAACACCTATGGAAGC-3’. The PCR products were purified and sequenced at the DNA Sequencing Core of Brigham and Women’s Hospital. The sequencing results were compared with mouse and human CRTAC1 mRNA sequences from NCBI.

### Micro-CT Scan

To compare the trabecular bone parameters of wild type and *Crtac1* knockout mice, distal femurs were scanned using a Scanco μCT-35 at 7 micron (μm) resolution. Morphometric analysis at the distal femoral end began at the growth plate, and extended proximally through the metaphysis for 300 slices. Bone volume fraction (BV/TV), trabecular number (Tb. N [1/mm]), trabecular thickness (Tb.Th [mm]), and trabecular separation/spacing (Tb.Sp [mm]) were determined using software provided by the manufacturer. Cortical bone thickness was determined at the femoral midshaft. Thresholds of 220 and 400 were used for trabecular and cortical bone analyses, respectively.

### Statistical analysis

All data are presented as mean ± SD. Differences between two groups were evaluated by two-tailed Student’s t test. For histological scorings of mouse OA, comparison between groups was performed using the nonparametric Mann-Whitney test. Two-Way ANOVA followed by Sidak’s test for multiple comparisons was performed as indicated. All analyses were performed using Prism 6.0 (GraphPad). P value < 0.05 was considered significant.

## Results

### Upregulation of CRTAC1 in the articular cartilage of human OA

CRTAC1 is a cartilage-derived protein. Microarray analysis identified an increase in the expression of *CRTAC1* mRNA in human OA articular cartilage [10, 11]. Real-time PCR and IHC were used to validate this finding. First, a higher level of *CRTAC1* mRNA was found in lesional vs. macroscopically normal cartilage biopsies from patients undergoing total knee replacement (Fig 1B). Likewise, CRTAC1 IHC displayed more intense staining and more numerous CRTAC1-positive cells in lesional compared to non-lesional articular cartilage (Fig 1C). These CRTAC1-positive cells mainly located in the superficial and upper intermediate layers of the lesional articular cartilage, but not in the calcified cartilage layer. Together, these results confirm the upregulation of CRTAC1 in the articular cartilage of human OA.

### IL-1β and TNF-α stimulate CRTAC1 expression in human articular chondrocytes and synovial fibroblasts

**Fig 2:**
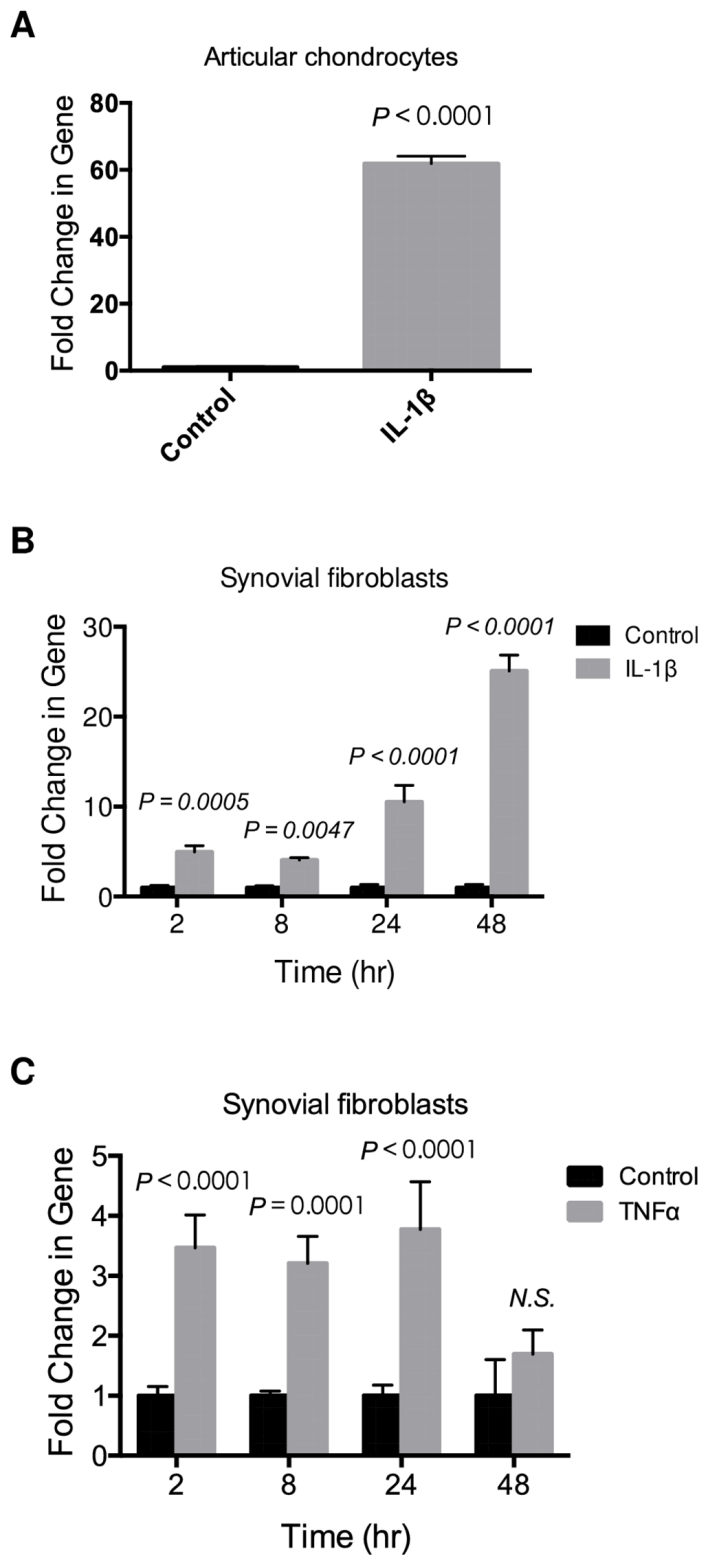
IL-1β and TNF-α stimulate CRTAC1 expression in human articular chondrocytes and synovial fibroblasts. **A**, Real-time PCR analysis of *CRTAC1* expression in human articular chondrocytes isolated from macroscopically normal parts of OA cartilage treated by IL-1β for 24 hrs. Two-tailed *t-test* was performed. **B**, Real-time PCR analysis of *CRTAC1* expression in human OA synovial fibroblasts after treatment with IL-1β for indicated times. **C**, Real-time PCR analysis of *CRTAC1* in human OA synovial fibroblasts treated with TNF-α for indicated times. Two-Way ANOVA followed by Sidak’s test for multiple comparisons was performed for **B** and **C**. All data are presented as means ± SDs.

Proinflammatory cytokines like IL-1β and TNF-α likely play an important role in the pathophysiology of OA and upregulate many of the catabolic processes that contribute to cartilage degradation [28–30]. Interestingly, IL-1β dramatically upregulated *CRTAC1* in articular chondrocytes (Fig 2A). Since transcriptomic analysis of human OA synovium also displayed an increase of *CRTAC1* [10, 12], the expression of this gene was measured in OA synovial fibroblasts treated with IL-1β or TNF-α. Both cytokines induced *CRTAC1* mRNA in OA synovial fibroblasts within two hours (Fig 2B and 2C). Expression of CRTAC1 continued to increase in response to IL-1β over 48 hours. In contrast, expression in response to TNF-α plateaued at 2 hours and did not increase further with time. These data indicate that the proinflammatory cytokines IL-1β and TNF-α directly induce the expression of CRTAC1 in articular chondrocytes and synovial fibroblasts.

### Expression of *Crtac1* in mouse articular cartilage

*Crtac1* is upregulated in the articular cartilage of mice with post-traumatic OA [13]. Thus, we sought to define the role of this gene in the pathogenesis of OA using genetically modified mice. Previous studies suggest that only CRTAC1B is expressed in mouse [15]. To identify the isoform of *Crtac1* expressed in mouse articular cartilage, we performed RACE to amplify the 3′-end of mouse *Crtac1* mRNA (S1 Fig), since the main difference between *CRTAC1A* and *CRTAC1B* resides in the sequence of the last exon (exon 15) in humans. By nested amplification using two gene-specific primers, the PCR products displayed two main bands in both mouse brain and articular cartilage tissue at sizes of ~800 and 300 bp, respectively (S1 Fig). Sequencing indicated that both bands matched *mus*. *musculus cartilage acidic protein 1* (*Crtac1*) (NCBI reference sequence: NM_145123.4), and were homologous to human CRTAC1B (S1 and S2 Figs). The difference in length of the two bands lies in the length of the 3’ UTR without affecting the coding sequence. Instead, difference in the 3’UTR may reflect alternative cleavage and polyadenylation at the 3’ end of mouse *Crtac1* mRNA, which may affect the mRNA localization, translation and degradation [31, 32]. Thus, our results provide direct evidence that mouse articular cartilage expresses the homologous isoform of human *CRTAC1B*.

### *Crtac1* knockout mice demonstrate normal bone and cartilage development at 12 weeks of age

**Fig 3:**
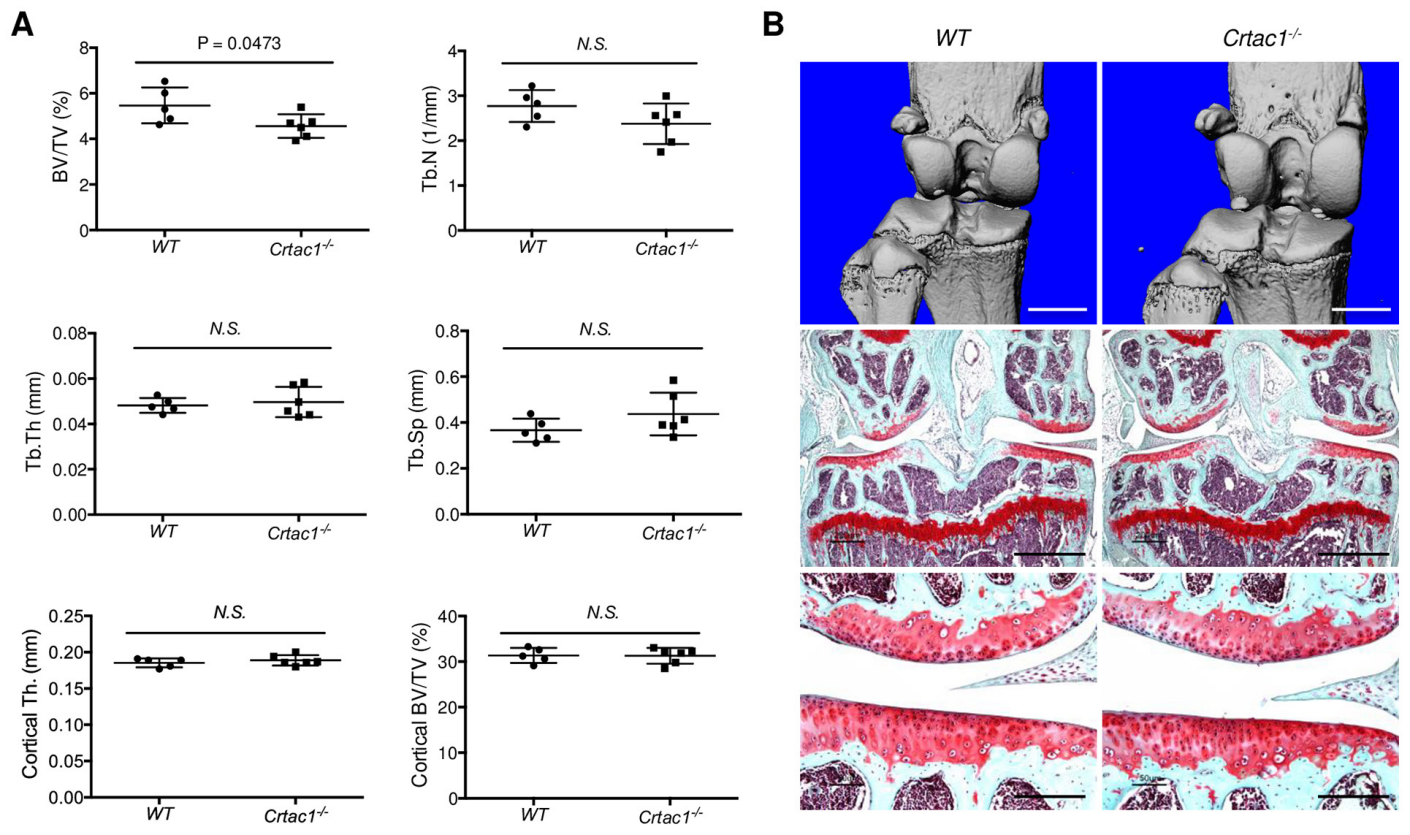
*Crtac1* knockout mice demonstrate normal bone and cartilage development at 12 weeks of age. **A**, Micro-CT (μCT) analyses of trabecular (BV/TV, Tb.N, Tb.Th, Tb.Sp) and cortical (Cortical Th., Cortical BV/TV) bone parameters in female wild type and *Crtac1*^*-/-*^ mice of 12 weeks age. All data are means ± SDs. Two-tailed *t-tests* were performed. **B**, Three-D μCT images (upper panels) and safranin-O/fast green stain (middle and lower panels) of mouse knee joints showing bone or articular cartilage of wild type and *Crtac1*^*-/-*^ female mice at 12 weeks of age. The local lack of safranin-O staining in articular cartilage of the femoral condyle of *Crtac1*^*-/-*^ mouse is an artifact of cutting/staining, not a real lesion. The artifact is also observed in the femoral condyle of WT mouse in S3B Fig. Scale bars, 1 mm (upper panels), 800 μm (middle panels), 200 μm (lower panels).

To exclude developmental skeletal defects that could influence surgically induced OA, the baseline bone and articular cartilage phenotype of 12 weeks old *Crtac1* knockout mice was analyzed. Ex vivo μCT analyses showed a statistically significant reduction in trabecular BV/TV in female *Crtac1*^*-/-*^ mice (Fig 3A). However, the magnitude of this difference was only 16.6%. In contrast, no differences were found in trabecular number, thickness or spacing, or cortical bone parameters between female wild type and *Crtac1*^*-/-*^ mice (Fig 3A). All bone parameters were similar between male wild type and *Crtac1*^*-/-*^ mice (S3 Fig). No baseline histologic abnormalities were detected in the knee articular cartilage of either female or male knockout mice (Fig 3B and S3 Fig). Furthermore, these *Crtac1* knockout mice demonstrated a normal knee articular cartilage at the age of 26 weeks (S4 Fig). Thus, the lack of a significant baseline skeletal phenotype in *Crtac1*^*-/-*^ mice make them a useful model to explore the role of this gene in post-traumatic OA.

### Deletion of CRTAC1 inhibits post-traumatic OA in female mice

**Fig 4:**
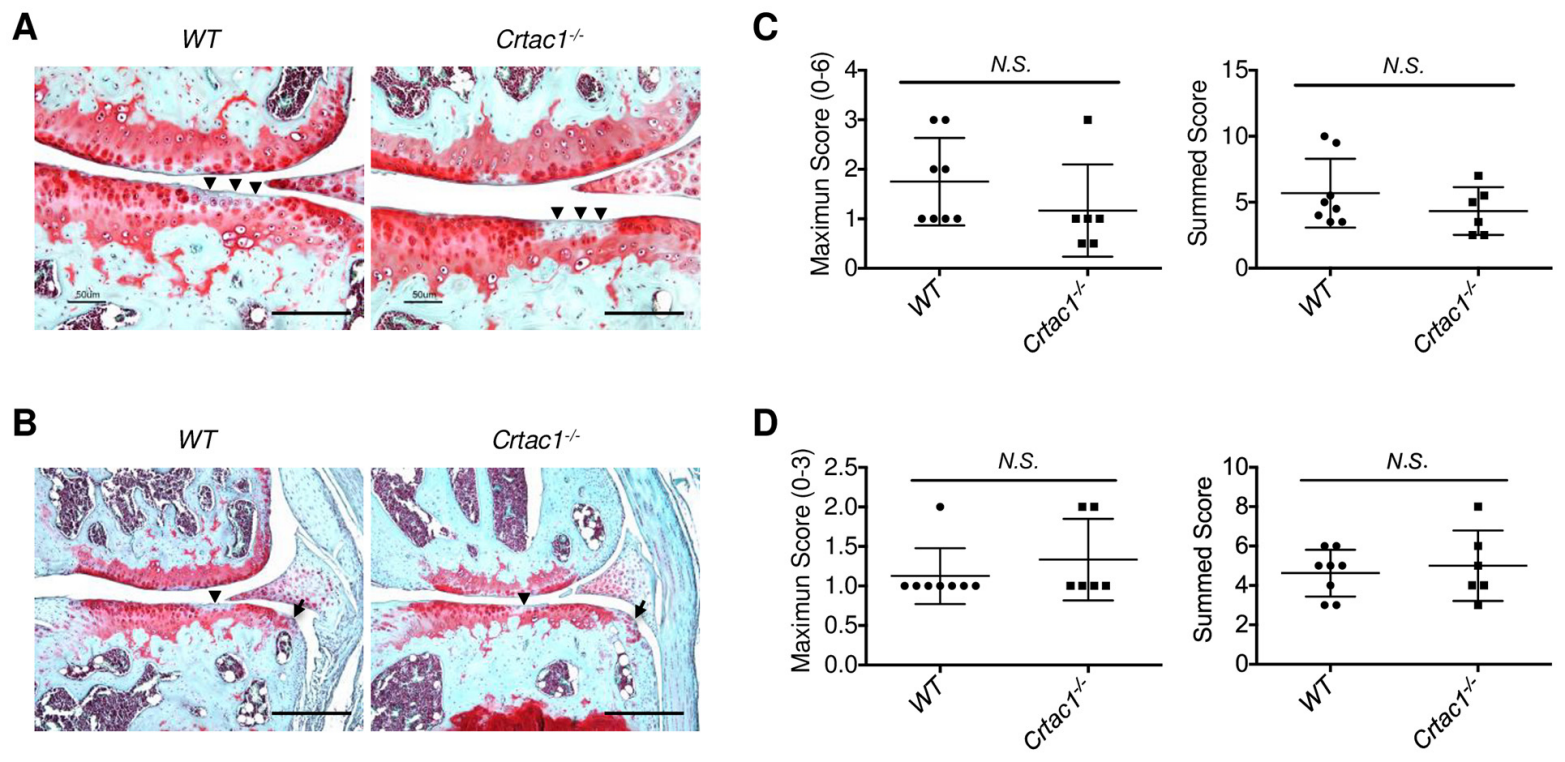
Similar histologic changes of OA in wild type and *Crtac1*^*-/-*^ mice 8 weeks after DMM surgery. **A** and **B**, Safranin-O/fast green stain of right knee joints of wild type and *Crtac1*^*-/-*^ mice showing changes of articular cartilage (arrowheads) and osteophyte formation (**B**, arrows) 8 weeks after DMM surgery. **C** and **D**, The maximum and summed OA histologic scores for articular cartilage (**C**) and osteophytes (**D**) in wild type and *Crtac1*^*-/-*^ mice 8 weeks after DMM surgery. Data were pooled from male (5 wild type, 3 *Crtac1*^*-/-*^) and female (3 wild type, 3 *Crtac1*^*-/-*^) animals. The maximum cartilage scores for male mice: 5 WT: 1, 1, 2, 2, 3; 3 *Crtac1*^*-/-*^: 0.5, 1, 3; and for females: 3 WT: 1, 1, 3; 3 *Crtac1*^*-/-*^: 0.5, 1, 1. The summed cartilage scores for male mice: 5 WT: 3.5, 4.5, 5, 5.5, 10; 3 *Crtac1*^*-/-*^: 2.5, 5.5, 7; and for females: 3 WT: 3.5, 4, 9.5; 3 *Crtac1*^*-/-*^: 2.5, 3.5, 5. The maximum osteophyte scores for male mice: 5 WT: 1, 1, 1, 1, 2; 3 *Crtac1*^*-/-*^: 1, 1, 2; and for females: 3 WT: 1, 1, 1; 3 *Crtac1*^*-/-*^: 1, 1, 2. The summed osteophyte scores for male mice: 5 WT: 3, 3, 5, 6, 6; 3 *Crtac1*^*-/-*^: 3, 4, 8; and for females: 3 WT: 4, 5, 5; 3 *Crtac1*^*-/-*^: 4, 5, 6. All data are means ± SDs. Nonparametric Mann-Whitney tests were performed. Scale bars: 200 μm (**A**), 400 μm (**B**).

**Fig 5:**
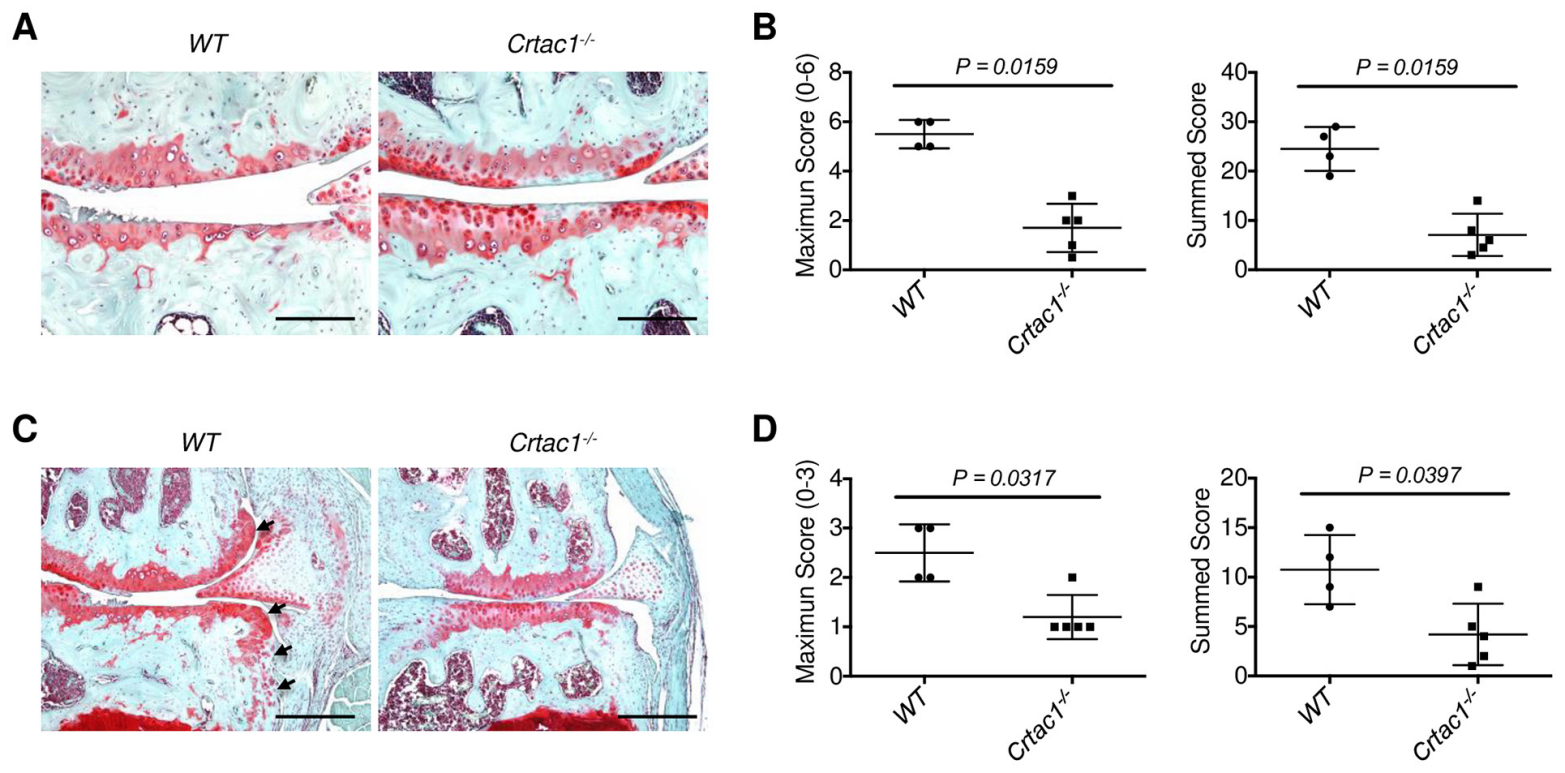
Ablation of CRTAC1 inhibits post-traumatic OA in female mice. **A**, Safranin-O/fast green stain of knee articular cartilage of wild type and *Crtac1*^*-/-*^ female mice 16 weeks after DMM surgery showing decreased structural destruction of articular cartilage after deletion of *Crtac1*. **B**, The maximum and summed OA histologic scores for articular cartilage 16 weeks after DMM surgery in wild type and *Crtac1*^*-/-*^ female mice. **C**, Safranin-O/fast green stain of right knee joints showing that ablation of *Crtac1* reduce osteophyte formation at the margin of articular cartilage (arrows) 16 weeks after DMM surgery. **D**, Maximum and summed histologic scores of osteophytes in wild type and *Crtac1*^*-/-*^ female mice 16 weeks after DMM surgery. Scale bars: 200 μm (A), 400 μm (C). All data are means ± SDs. Nonparametric Mann-Whitney tests were performed.

The mouse DMM model of OA faithfully recapitulates the natural progression of human OA [20, 33, 34]. DMM surgery was performed on male and female *Crtac1*^*-/-*^ mice and wild type littermate controls, and structural deterioration of the knee joints was examined 8 and 16 weeks later by histology. Eight weeks after surgery, similar early features of OA were evident in *Crtac1*^*-/-*^ and wild type mice, including loss of safranin-O staining, fibrillation of the articular surface and mild destruction of articular cartilage (Fig 4A). Accordingly, histologic scores for articular cartilage were similar at this time point (Fig 4B). Likewise, both wild type and *Crtac1*^*-/-*^ animals displayed mild osteophyte formation at this time point (Fig 4C and 4D). Strikingly, compared to 8 weeks after DMM surgery, at 16 weeks wild type mice demonstrated progressive, severe cartilage destruction and osteophyte formation. In contrast, female mice lacking *Crtac1* failed to progress further with cartilage destruction and osteophyte formation essentially arresting between these two time points (Fig 5A–5D). These data suggest that CRTAC1 is involved in OA progression rather than in the initiation of disease. A second observer who scored the sections independently confirmed these findings (S5 Fig). Intriguingly, protection from DMM induced OA was only found in female mice, as male *Crtac1*^*-/-*^ mice and wild-type controls did not display significant difference in histologic OA scores (S6 Fig).

### Deletion of CRTAC1 inhibits OA related gait abnormalities in female mice

**Fig 6:**
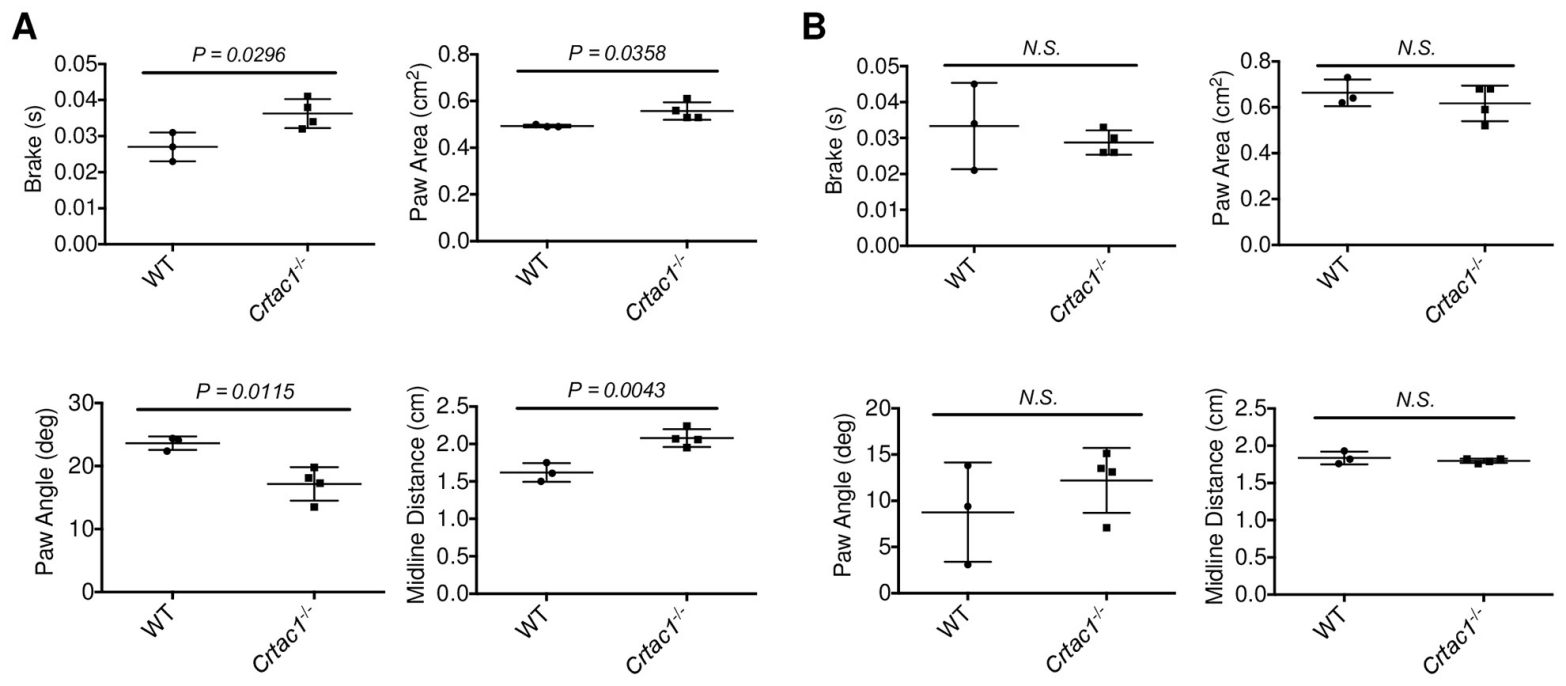
Deletion of CRTAC1 inhibits pain of post-traumatic OA in female mice. **A**, Gait analyses of right hind limb (surgical) of wild type and *Crtac1*^*-/-*^ female mice 16 weeks after DMM surgery displaying decreased brake time and paw angle, and increased paw area and midline distance after ablation of *Crtac1*. **B**, Gait analyses showing no significant difference about these parameters in contralateral left hind limb of wild type and *Crtac1*^*-/-*^ female mice 16 weeks after DMM surgery. All data are means ± SDs. Two-tailed *t-tests* were performed.

Gait analysis has been used to measure the pain related behaviors in rodent OA models [35, 36]. Compared to controls, female *Crtac1*^*-/-*^ mice displayed increased paw area and brake time of right hindlimb (surgical), suggesting a reduction in pain with weight bearing (Fig 6A). In addition, a decreased paw angle and increased midline distance was also observed in female *Crtac1*^*-/-*^ mice compared to wild type controls, indicating greater control and better reach of the right hind limb in *Crtac1*^*-/-*^ mice, respectively. In contrast, no significant differences were observed in gait parameters of the contralateral left hind limb (Fig 6B). Taken together, these results indicate that deletion of *Crtac1* protects female mice from both the structural and gait manifestations of post-traumatic OA.

## Discussion

In this study, we identify CRTAC1 as a novel regulator of OA pathogenesis. This gene is upregulated in the SF, cartilage and synovium of patients with OA and mice with post-traumatic OA. *CRTAC1* is induced by pro-inflammatory cytokines in articular chondrocytes and synovial fibroblasts and deletion of this gene in female mice inhibits the development of OA. This female-specific suppression of OA by deleting CRTAC1 is particularly meaningful, considering that the incidence and severity of human OA are significantly higher in women than men [3–5].

By comparing the expression of CRTAC1 in lesional versus non-lesional foci in human OA cartilage, we found a significant increase of CRTAC1 expression in the lesional articular cartilage. It should be highlighted that the non-lesional articular cartilage from OA patients may display early changes of OA such as fibrillation of cartilage surface and/or loss of cartilage extracellular matrix proteoglycan, or other biochemical abnormalities, and thus is not an ideal control. However, obtaining age, gender and excise matched normal human articular cartilage is extremely challenging. Therefore, our data are helpful for confirmation of CRTAC1 expression in degrading cartilage during OA. Moreover, it confirms our previous study where, through the use of gene expression profiling, elevated levels of *CRTAC1* mRNA were identified in OA cartilage and synovium compared to normal controls [10].

Immunohistochemical results displayed that CRTAC1-positive cells mainly locate in the superficial and upper intermediate layers of the lesional OA cartilage, but not in the calcified cartilage layer. This is different from a previous report by Steck et al., which showed that CRTAC1 is mainly expressed in the calcified articular cartilage [15]. The difference may result from the different cartilage tissue used for the immunostaining. While Steck et al. used the cartilage tissue from a hypoplasic thumb of a 6-year-old boy, our study used the late OA cartilage from adults. Although they reported a result of in situ hybridization for *CRTAC1* using articular cartilage from a knee OA patient, their reliance on in situ hybridization on decalcified tissue and lack of immunostaining for this tissue make their study difficult to compare with our results. In addition, the specificity of antibodies used in the two studies may also contribute to the difference of CRTAC1 staining. In a pilot study, another CRTAC1 antibody (from GeneTex) was also tested in our study. These two different antibodies generated similar expression pattern of CRTAC1 in human OA cartilage. However, the antibody from R&D, which was reported in this study, demonstrated a lower background of staining. Thus, by using multiple human OA samples (n = 6), testing lesional and non-lesional samples and verifying our results with two different CRTAC1 antibodies, our results provide convincing evidence that chondrocytes in the superficial and upper intermediate layers are the main source of enhanced CRTAC1 in OA articular cartilage.

IL-1β and TNF-α are two major proinflammatory cytokines in the initiation and progression of OA [37, 38]. They are secreted by articular chondrocytes, synovial cells and immune cells in articular tissues and display elevated levels in OA SF. These proinflammatory cytokines promote catabolism and inhibit anabolism of articular cartilage, and lead to inflammation during OA. In the present study, our data showed that IL-1β and TNF-α significantly upregulate the expression of *CRTAC1* in articular chondrocytes or synovial fibroblasts, suggesting proinflammatory cytokines could be the upstream signaling causing enhanced CRTAC1 in OA. Previous reports showed that upregulation of IL-1β and TNF-α in human OA cartilage is preferentially located in the superficial and upper intermediate layers of articular cartilage [29, 39, 40], demonstrating a similar distribution pattern to CRTAC1 in our study.

How CRTAC1 affects OA progression remains unclear. There are at least four possible mechanisms we can envision. First, since our results show that IL-1β and TNF-α induce CRTAC1 expression in human articular chondrocytes and synovial fibroblasts, CRTAC1 may mediate the effects of cytokines during OA, including promoting the catabolic and inhibiting the anabolic activities of chondrocytes. Second, human CRTAC1 in the articular cartilage is a glycosylated extracellular matrix protein [15]. Upregulation of this protein in the articular cartilage of human OA might disturb matrix turnover and cartilage homeostasis, and thus alter the biophysical properties of cartilage extracellular matrix, e.g. its stiffness. These changes could further affect the cellular behaviors of chondrocytes to intensify OA progression [41–43]. Third, increased levels of CRTAC1 in OA SF and articular cartilage might be able to affect the remodeling of the subchondral bone. A recent paper showed that the downstream target of CRTAC1, NOGO-A/NGR1 pathway, is necessary for osteoclastogenesis [44]. Thus, elevated CRTAC1 in OA may antagonize NOGO/NGR1 signaling to inhibit osteoclast formation and promote subchondral bone sclerosis, which could exacerbate OA progression [45, 46]. Lastly, the NOGO-A/NGR1 signaling pathway inhibits axon outgrowth and regeneration [18, 47]. Since pain is a main symptom of OA, increased expression of CRTAC1 may impair NGR1 mediated axon growth inhibition and promote the neuronal growth in the OA synovium and subchondral bone to enhance pain. Consistent with this, gait analysis results show that *Crtac1* knockout mice display less pain after the induction of posttraumatic OA.

The molecular understanding of the increased prevalence and severity of knee OA in women remains poorly understood. Here, we identify CRTAC1 as a novel molecule that specifically boosts OA progression in female animals. In addition to sex hormones and genes on sex chromosomes, genes on autosomes could also result in the gender differences of diseases by an underlying mechanism of regulatory genome [48]. It should be noted that although the sex differences regarding CRTAC1 expression was not carefully examined in previous human proteomic and transcriptomic data, this gene is likely upregulated in both genders during OA, especially since the transcriptomic data showing an increase of *Crtac1* in mouse OA was acquired using male mice [13]. Furthermore, even in the absence of the gender differences regarding gene expression, a genotype-sex interaction can still occur with respect to a disease phenotype, both in OA and other diseases influenced by sex, like hypertension [48–50]. Interesting, a recent study showed that female mice lacking *Ngr1*, the downstream target of CRTAC1, displayed greater extinction of tone-associated fear conditioning than male animals [51]. Thus, further study will be necessary to better define the mechanism underlying the gender-specific role of CRTAC1 in OA and whether this involves the interaction of CRTAC1 with NGR1 or other pathways, e.g. gonadal steroids or additional sex-dependent molecules.

It is noteworthy that the isoform of *Crtac1* expressed in mice (*Crtac1b*) is predicted to encode a C-terminal transmembrane domain. Thus, CRTAC1B is likely membrane surface protein [16, 17]. In contrast, human articular chondrocytes express *CRTAC1A*, which lacks a transmembrane domain and is secreted. Although CRTAC1A and CRTAC1B share homologous functional protein domains and are upregulated in human and mouse OA, respectively, they may function differentially based on the soluble or transmembrane localization. Alternatively, mouse *Crtac1b* might be secreted or cleaved, and thus represent functional homologue of human CRTAC1A. Additional biochemical characterization will be needed to decipher the cellular and tissue localization of CRTAC1A and CRTAC1B, and whether they function similarly to promote OA in mouse and man. Furthermore, it is also unknown whether the different isoforms of CRTAC1 in humans and mice affect the gender-specific effect of this molecule on OA progression.

There are some limitations to the present study. First, a small sample size (n = 3–5) was used for female or male mice at each time point. This may explain why we did not observe a significant difference of histologic scores between female wild type and *Crtac1*^*-/-*^ mice 8 weeks after DMM surgery. Increasing the sample numbers may result in a statistically significant difference about the histology of OA in female animals at this time point. In addition, the baseline skeletal phenotypes of female *Crtac1*^*-/-*^ mice demonstrate a slight decrease of trabecular BV/TV at 12 weeks of age. As discussed above, this may result from a potential inhibitory effect of CRTAC1 on osteoclastogenesis. Ablation of this gene may lead to increased osteoclast formation and thereby a reduction of trabecular BV/TV. However, this absolute difference in bone mass is mild (about 16.6%), and these animals do not demonstrate other skeletal abnormalities, especially about the articular cartilage. Thus, we would not expect the decreased trabecular BV/TV to directly affect the progression of DMM induced OA in these animals. Lastly, we did not acquire the gait parameters for male mice because of the limited availability of the gait analysis machine. However, a difference in gait parameters would not be expected between the male WT and *Crtac1*^*-/-*^ mice based on no significant difference in histology 16 weeks after surgery. Future studies exploring the mechanism of CRTAC1 in OA progression should carefully examine whether CRTAC1 holds a role in OA-related knee pain independent of joint structure deterioration.

The mouse DMM model has been widely used for preclinical studies, which leads to mild to moderate OA [20, 33, 34]. However, significant variability has been reported in this model [52]. This may make studies using this model challenging to interpret and limit their validity. The variability in the DMM model may be related to differences in surgical technique among investigators, the time points used for histologic evaluation, histologic scoring methods and stochastic variation among individual animals. We have made efforts to address each of the potential sources of inconsistency. Based on our experience, the trauma of surgery does affect the severity of subsequent OA. Trauma could result in damage to joint structures other than the medial meniscal ligament (e.g. joint capsule, articular cartilage, intraarticular fat pat, meniscus) as well as inflammation. Thus, surgical trauma was minimized by reducing exposure of joint cavity and damage to the patellar ligament, joint capsule and fat pat, avoiding damage to articular cartilage, as well as carefully cleaning the wound, closing joint cavity and repositioning tissues. Our experience using C57/BL6 mice suggests that mice develop early or modest manifestation of OA 8 weeks after surgery, including loss of safranin-O stain, fibrillation of articular surface or mild destruction of articular cartilage (maximum histologic score ≤ 3). Sixteen weeks after DMM surgery these mice develop severe destruction of articular cartilage (maximum histologic score ≥ 3), formation of osteophytes and subchondral bone sclerosis. Although we did not observe dramatic individual variability at these time points, the male mice tended to have greater variability than female animals. Lastly, two blinded observers were utilized to score our histology and produced similar results. Taken together, our rigorous approach to the execution and interpretation of the DMM OA model make it unlikely that the protection from post-traumatic OA we observed in female *Crtac1*^*-/-*^ mice was due to chance.

In summary, CRTAC1 is an important regulator of OA pathogenesis. Inhibition of this gene/protein may reduce OA specifically in females. Future studies should seek to better understand the gender—specific effects of CRTAC1 in OA and to identify small molecules or antibodies that block CRTAC1 which could be used to treat OA.

## Supporting Information

S1 Fig Direct amplification of 3′-end (exon 15) of mouse *Crtac1* by classic rapid amplification of cDNA ends (RACE). **A**, schematic showing the approximate binding locations of gene specific primer (GSP) 1 and 2, as well as the primer at the 3′ end containing a 17 nucleotide oligo-(dT) sequence on the mouse *Crtac1* mRNA. **B**, Gel image of RACE PCR products after nested amplification of cDNA prepared from mouse brain (MB) and articular cartilage (AC) showing long (~800bp) and short (~300bp) bands. The PCR amplification reaction without cDNA was used as the negative control (Neg). **C**, Parts of sequences of the long and short bands by sequencing the purified RACE PCR products from **B**. “TAA” in red square in the sequence of long band represents stop codon of mouse *Crtac1* mRNA. Arrow indicates the beginning of exon 15 in the sequence of short band.
(PDF)

S2 Fig Comparison of the sequence of the *Crtac1* 3′ RACE products generated in S1 Fig with that of exons 14 and 15 of mouse *Crtac1* mRNA from NCBI. The sequence of the long (**A**) and short (**B**) bands map to exon 15 of mouse *Crtac1* (NCBI Reference Sequence: NM_145123.4). The sequence in **A** and **B** represents exons 14 and 15 (underlined) of mouse *Crtac1* mRNA. The blue sequence is the GSP2 for RACE, which was also used for sequencing the products. Green sequence represents the results of sequencing the long (**A**) and short (**B**) bands in S2B Fig. The red “TAA” sequence is the stop codon for *Crtac1* mRNA translation.
(PDF)

S3 Fig Normal skeletal development in male *Crtac1*^*-/-*^ mice at 12 weeks of age. **A**, Micro-CT (μCT) analyses of trabecular (BV/TV, Tb.N, Tb.Th, Tb.Sp) and cortical (Cortical Th., Cortical BV/TV) bone parameters in wild type and *Crtac1*^*-/-*^ male mice at 12 weeks of age. All data are means ± SDs. Two-tailed *t-tests* was performed. **B**, Three-D μCT images and safranin-O/fast green stain showing the knee joint morphology or articular cartilage of wild type and *Crtac1*^*-/-*^ male mice at 12 weeks of age. Images are representative of 5 mice. Scale bars: 1 mm (upper panels), 200 μm (lower panels).
(PDF)

S4 Fig Normal knee articular cartilage in female *Crtac1*^*-/-*^ mice at 26 weeks of age. Safranin-O/fast green stain showing the knee articular cartilage of wild type and *Crtac1*^*-/-*^ female mice at 26 weeks of age. Images are representative of 3 mice. Scale bars: 250 μm.
(PDF)

S5 Fig Deletion of *Crtac1* significantly inhibits post-traumatic OA in female mice. Maximum and summed histologic scores determined by a second blinded observer for articular cartilage (**A**) or osteophytes (**B**) on right knee joints of wild type and *Crtac1*^*-/-*^ female mice 16 weeks after DMM surgery. All data are means ± SDs. Nonparametric Mann-Whitney tests were performed.
(PDF)

S6 Fig Deletion of *Crtac1* does not affect OA progression in male mice. **A**, Safranin-O/fast green stain showing degradation of articular cartilage and osteophyte formation on right knees of wild type and *Crtac1*^*-/-*^ male mice 16 weeks after DMM surgery. Scale bars: 400 μm. **B**, The maximum and summed histologic scores for articular cartilage of wild type and *Crtac1*^*-/-*^ male mice 16 weeks after DMM surgery. Data are means ± SDs. Nonparametric Mann-Whitney tests were performed.
(PDF)
