# Supplementary figures and images for: Sex-Specific Protection of Osteoarthritis by Deleting Cartilage Acid Protein 1
Source: PLoS One. 2016 Jul 14;11(7):e0159157. doi: 10.1371/journal.pone.0159157 (PMC4945026; doi:10.1371/journal.pone.0159157)

**A**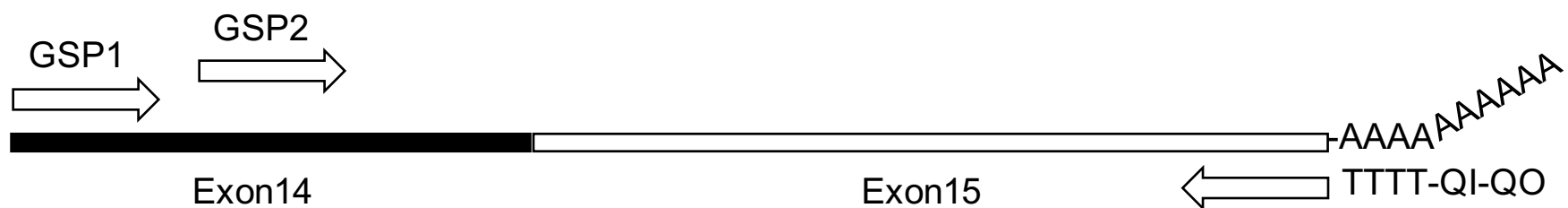**B**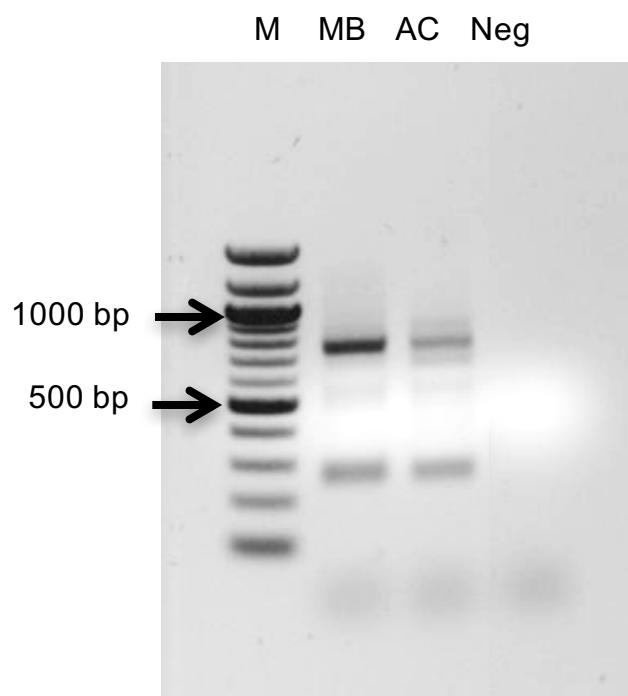**C**

Long band:

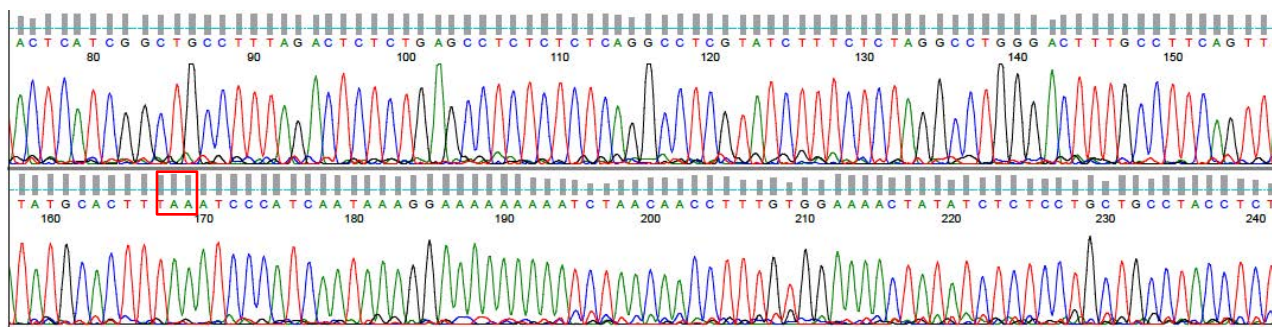

Short band:

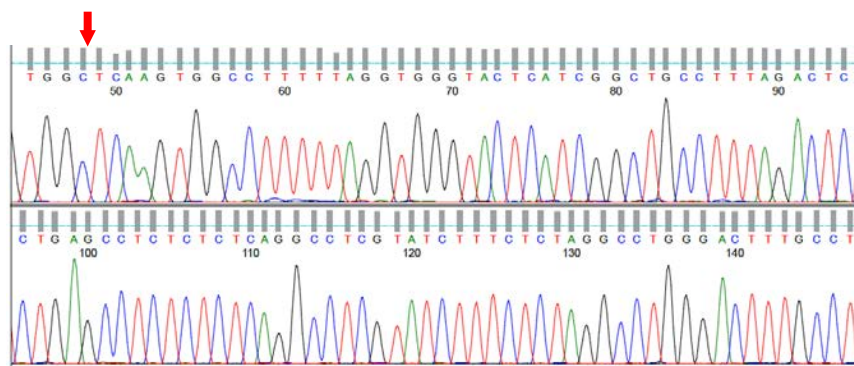

Supplement: S1 Fig — A, schematic showing the approximate binding locations of gene specific primer (GSP) 1 and 2, as well as the primer at the 3′ end containing a 17 nucleotide oligo-(dT) sequence on the mouse Crtac1 mRNA. B, Gel image of RACE PCR products after nested amplification of cDNA prepared from mouse brain (MB) and articular cartilage (AC) showing long (~800bp) and short (~300bp) bands. The PCR amplification reaction without cDNA was used as the negative control (Neg). C, Parts of sequences of the long and short bands by sequencing the purified RACE PCR products from B. “TAA” in red square in the sequence of long band represents stop codon of mouse Crtac1 mRNA. Arrow indicates the beginning of exon 15 in the sequence of short band. (PDF) [file pone.0159157.s001.pdf]

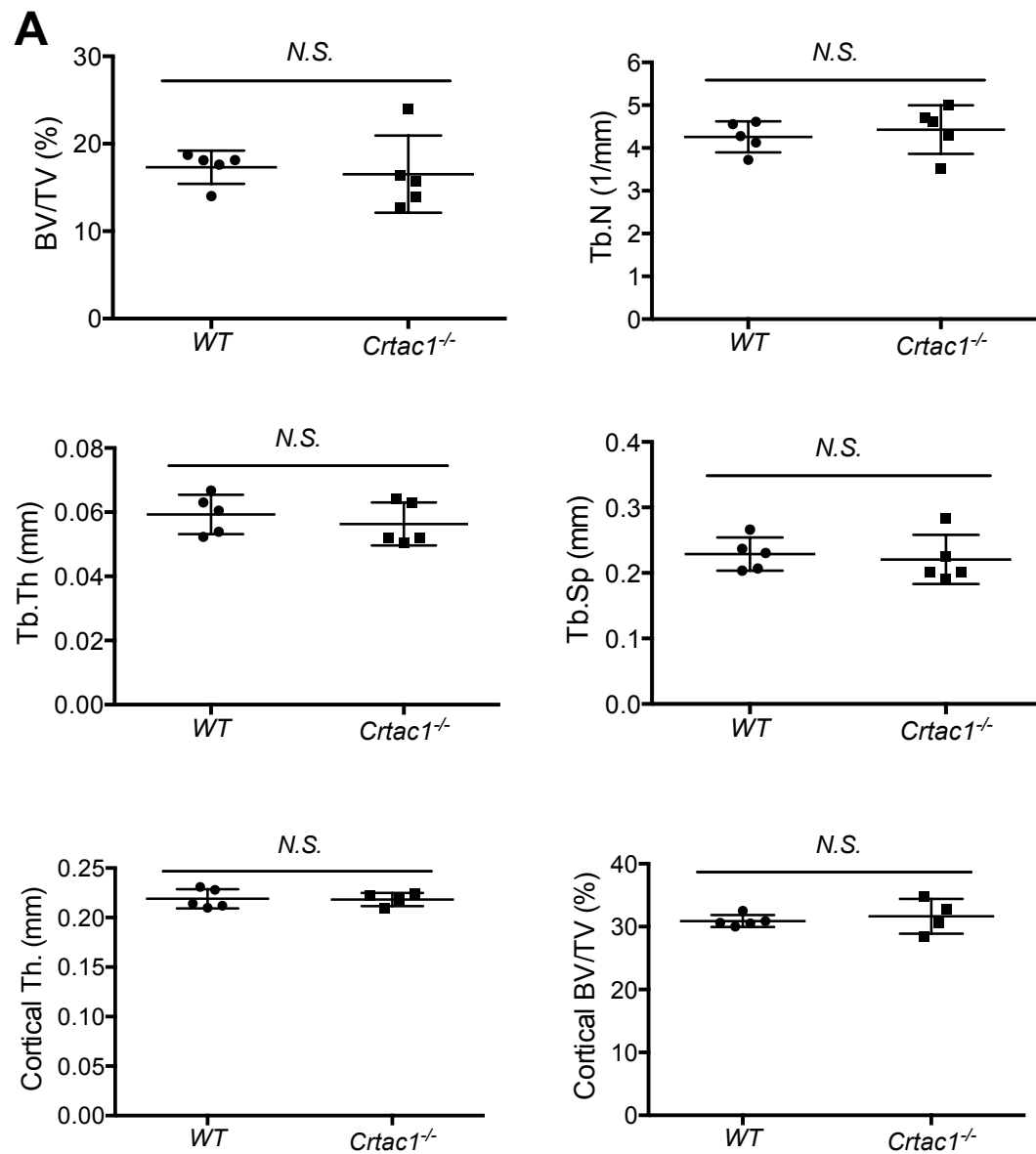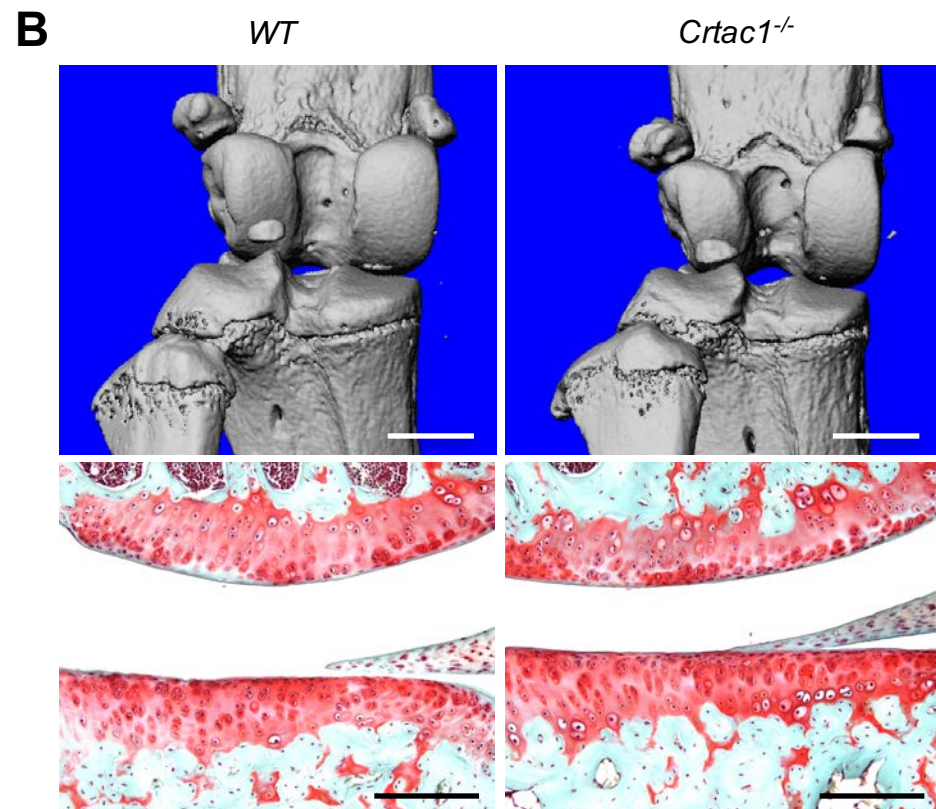

Supplement: S3 Fig — A, Micro-CT (μCT) analyses of trabecular (BV/TV, Tb.N, Tb.Th, Tb.Sp) and cortical (Cortical Th., Cortical BV/TV) bone parameters in wild type and Crtac1-/- male mice at 12 weeks of age. All data are means ± SDs. Two-tailed t-tests was performed. B, Three-D μCT images and safranin-O/fast green stain showing the knee joint morphology or articular cartilage of wild type and Crtac1-/- male mice at 12 weeks of age. Images are representative of 5 mice. Scale bars: 1 mm (upper panels), 200 μm (lower panels). (PDF) [file pone.0159157.s003.pdf]

*WT*

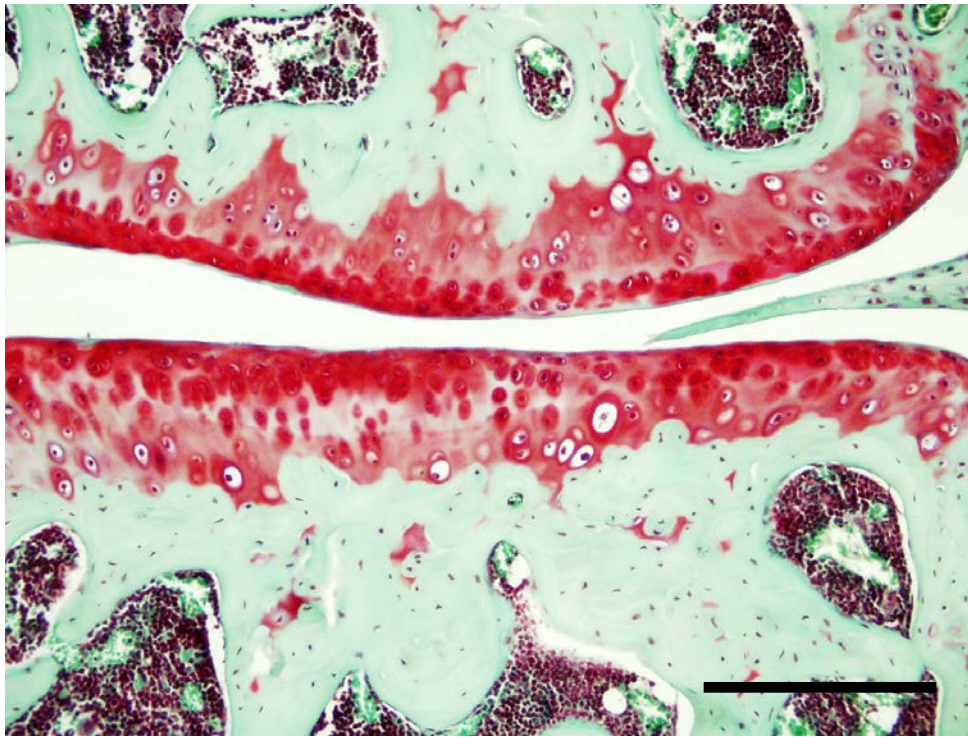

*Crtac1<sup>-/-</sup>*

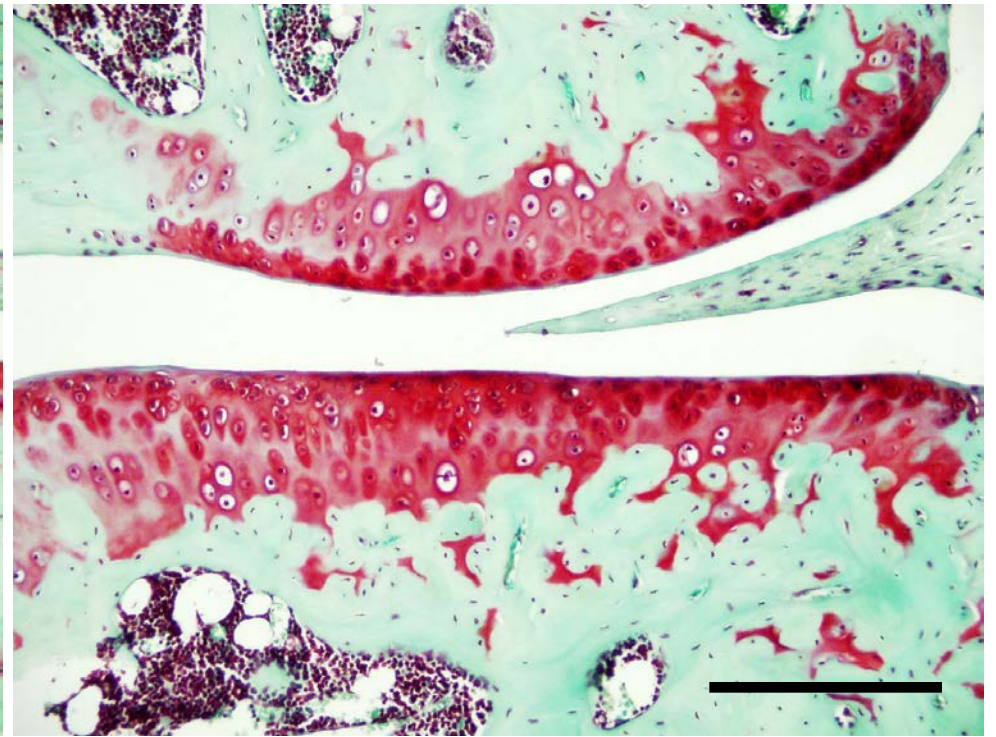

Supplement: S4 Fig — Safranin-O/fast green stain showing the knee articular cartilage of wild type and Crtac1-/- female mice at 26 weeks of age. Images are representative of 3 mice. Scale bars: 250 μm. (PDF) [file pone.0159157.s004.pdf]

**A**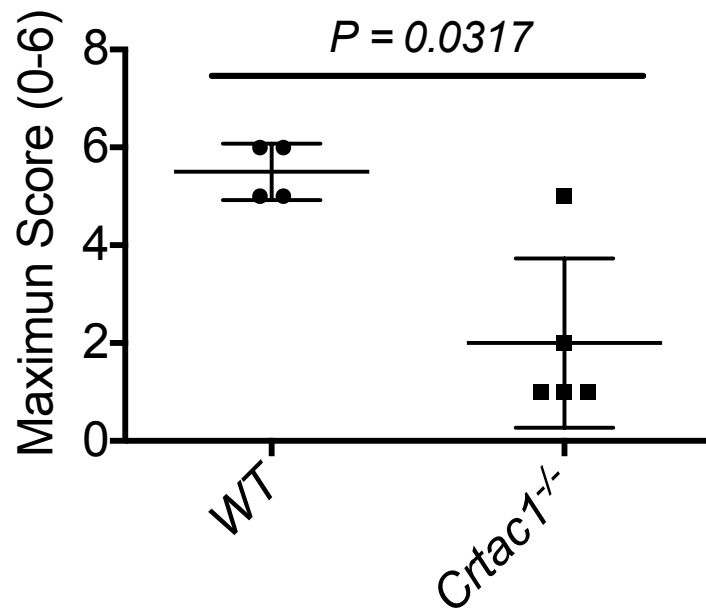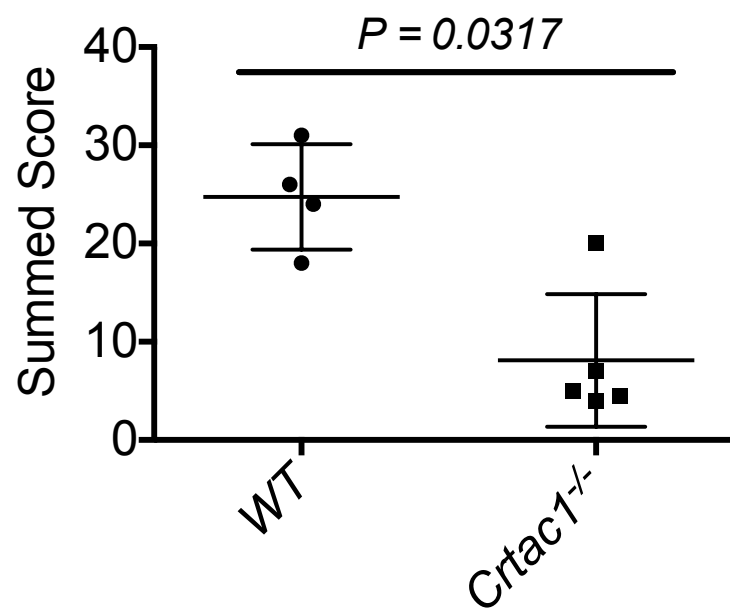**B**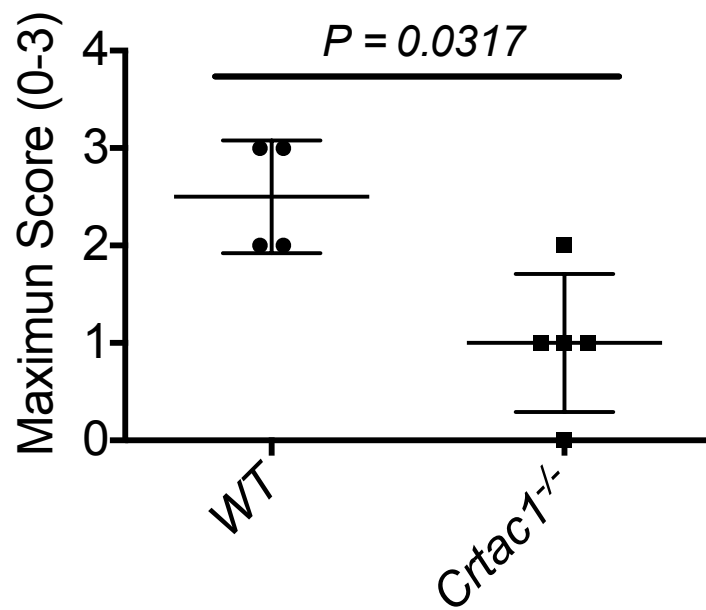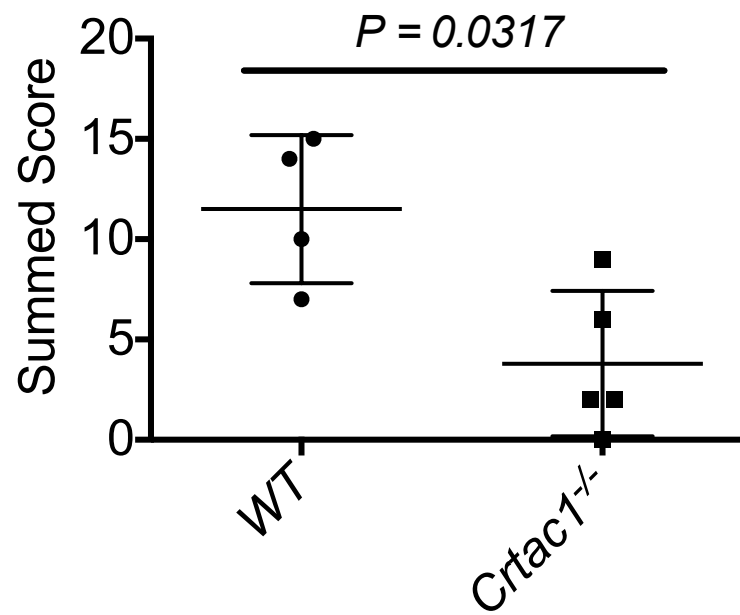

Supplement: S5 Fig — Maximum and summed histologic scores determined by a second blinded observer for articular cartilage (A) or osteophytes (B) on right knee joints of wild type and Crtac1-/- female mice 16 weeks after DMM surgery. All data are means ± SDs. Nonparametric Mann-Whitney tests were performed. (PDF) [file pone.0159157.s005.pdf]

**A***WT**Crtac1<sup>-/-</sup>*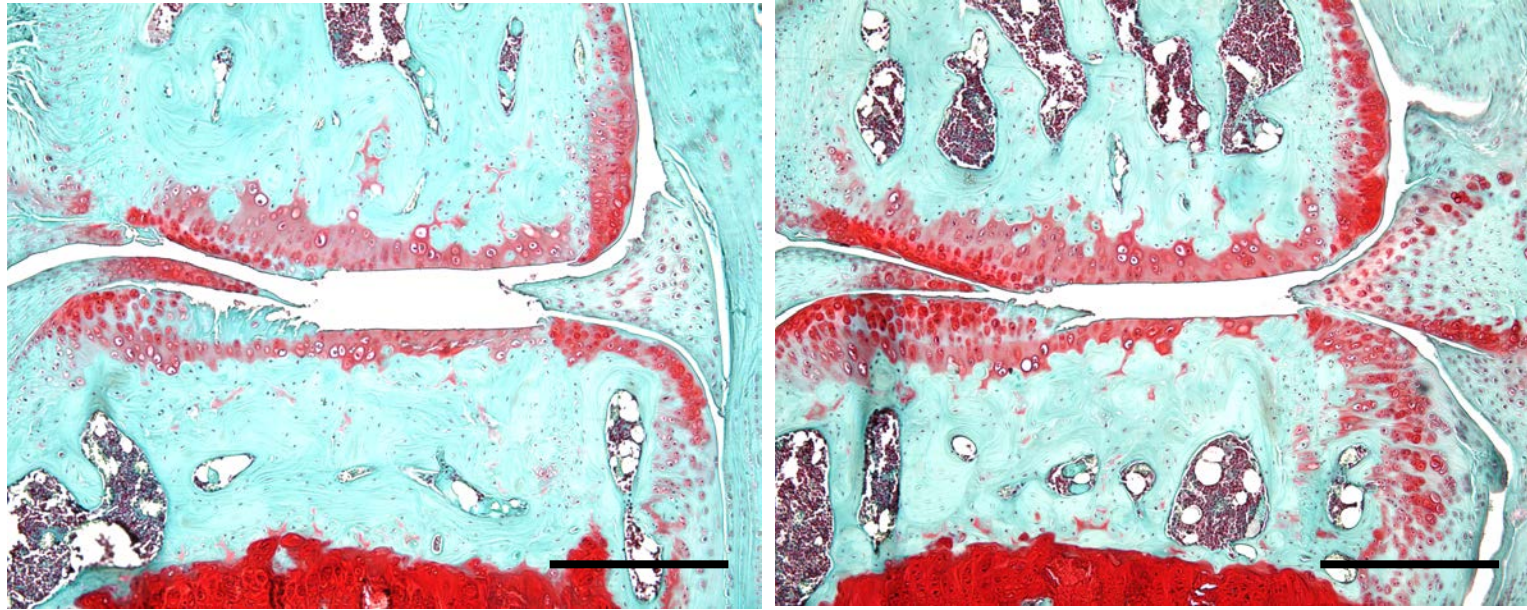**B**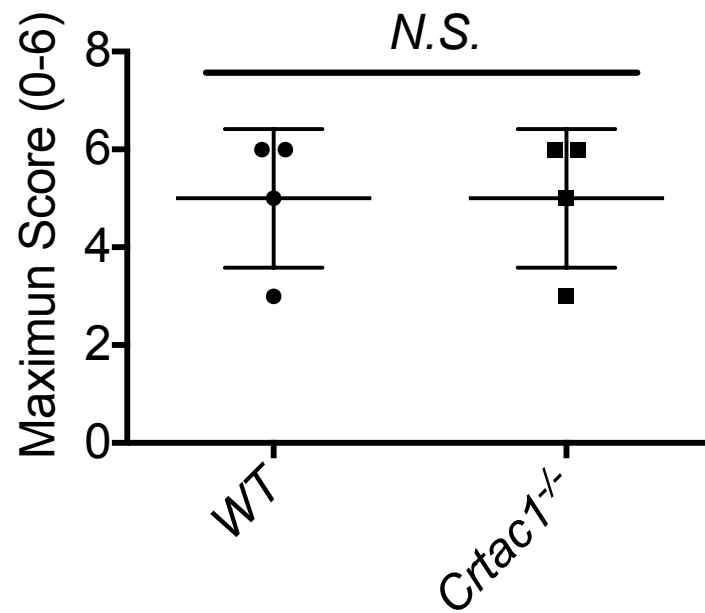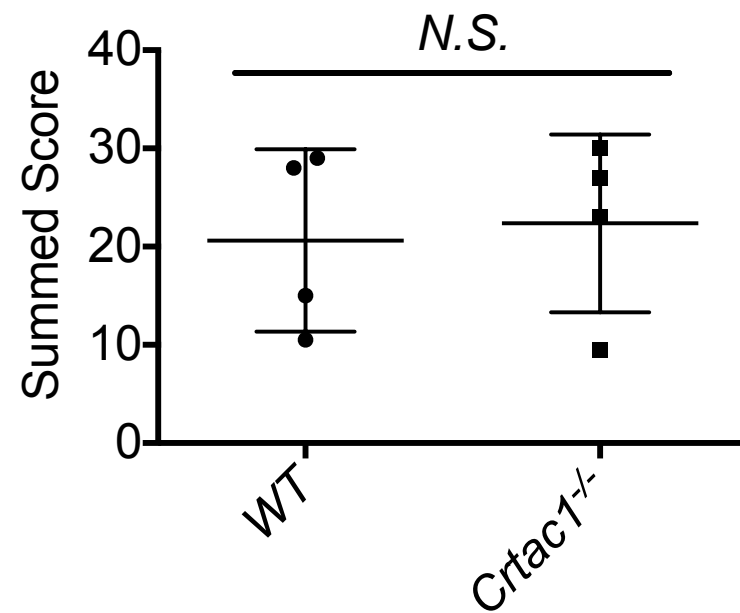

Supplement: S6 Fig — A, Safranin-O/fast green stain showing degradation of articular cartilage and osteophyte formation on right knees of wild type and Crtac1-/- male mice 16 weeks after DMM surgery. Scale bars: 400 μm. B, The maximum and summed histologic scores for articular cartilage of wild type and Crtac1-/- male mice 16 weeks after DMM surgery. Data are means ± SDs. Nonparametric Mann-Whitney tests were performed. (PDF) [file pone.0159157.s006.pdf]
