# Supplementary material for: Sex-Specific Protection of Osteoarthritis by Deleting Cartilage Acid Protein 1
Source: PLoS One. 2016 Jul 14;11(7):e0159157. doi: 10.1371/journal.pone.0159157 (PMC4945026; doi:10.1371/journal.pone.0159157)
Supplement: S2 Fig — The sequence of the long (A) and short (B) bands map to exon 15 of mouse Crtac1 (NCBI Reference Sequence: NM_145123.4). The sequence in A and B represents exons 14 and 15 (underlined) of mouse Crtac1 mRNA. The blue sequence is the GSP2 for RACE, which was also used for sequencing the products. Green sequence represents the results of sequencing the long (A) and short (B) bands in S2B Fig. The red “TAA” sequence is the stop codon for Crtac1 mRNA translation. (PDF) [file pone.0159157.s002.pdf]

Mus musculus cartilage acidic protein 1 (Crtac1), NCBI Reference Sequence: NM_145123.4

A

ACACCAATGAATGCATCCAGTTCCCATTTGTGTGCCCTCGAGACAAACCCGTATGTGTCAACACCTA
TGGAAGCTACAGGTGCCGGACCAATAAAAGATGCAATCGGGGCTATGAACCCAATGAAGACGGCA
CAGCCTGTGTGGCTCAAGTGGCCTTTTTAGGTGGGTACTCATCGGCTGCCTTTAGACTCTCTGAGC
CTCTCTCTCAGGCCTCGTATCTTTCTCTAGGCCTGGGACTTTGCCTTCAGTTATATGCACTTTAAATC
CCATCAATAAAGGAAAAAAAAATCTAACAACCTTTGTGGAAAACTATATCTCTCCTGCTGCCTACCTC
TCTCCACACCCCATAGATCCCGCCATGTGCTCCTTCGAACGGACAGCAGGGGTGGCCATCTTTGAA
AAGGGAAGTTTGGGAGATACTGATGGGACCTTAGTTGTTGGTGAAGCCTTTCTTTGCATGTTTTCTG
AGAAAACAGAAAAAGGAAAACTATCTCCTCTACCCCACCCCTCCTTCCGTGAGAAGAACCAAGAGA
GTCCAGTCATTTCTGTATCTTCGTGAGCTTCTTCTTGTTTTGCCCTTGACAAAGATGGCTCCTGGGT
GGTTGCTGGCTCGGTTGATTCAGTAATGTATCCTGTGCCTGCTGATCCGGCCTGTGCTGCTGCTGC
TGCTTTGTACAACCTGATTTCCTATGATTACGGAAGCCTTATTGGATTGGTGGTGGCGACGGAAAGG
GAAAGGGTGGCCCTTTGGGACTGTTGATAAAAAAAAAATGCTTAAGTTTGAACTT

B

ACACCAATGAATGCATCCAGTTCCCATTTGTGTGCCCTCGAGACAAACCCGTATGTGTCAACACCTA
TGGAAGCTACAGGTGCCGGACCAATAAAAGATGCAATCGGGGCTATGAACCCAATGAAGACGGCA
CAGCCTGTGTGGCTCAAGTGGCCTTTTTAGGTGGGTACTCATCGGCTGCCTTTAGACTCTCTGAGC
CTCTCTCTCAGGCCTCGTATCTTTCTCTAGGCCTGGGACTTTGCCTTCAGTTATATGCACTTTAAATC
CCATCAATAAAGGAAAAAAAAATCTAACAACCTTTGTGGAAAACTATATCTCTCCTGCTGCCTACCTC
TCTCCACACCCCATAGATCCCGCCATGTGCTCCTTCGAACGGACAGCAGGGGTGGCCATCTTTGAA
AAGGGAAGTTTGGGAGATACTGATGGGACCTTAGTTGTTGGTGAAGCCTTTCTTTGCATGTTTTCTG
AGAAAACAGAAAAAGGAAAACTATCTCCTCTACCCCACCCCTCCTTCCGTGAGAAGAACCAAGAGA
GTCCAGTCATTTCTGTATCTTCGTGAGCTTCTTCTTGTTTTGCCCTTGACAAAGATGGCTCCTGGGT
GGTTGCTGGCTCGGTTGATTCAGTAATGTATCCTGTGCCTGCTGATCCGGCCTGTGCTGCTGCTGC
TGCTTTGTACAACCTGATTTCCTATGATTACGGAAGCCTTATTGGATTGGTGGTGGCGACGGAAAGG
GAAAGGGTGGCCCTTTGGGACTGTTGATAAAAAAAAAATGCTTAAGTTTGAACTT
